# Multi-Scale Locality-Constrained Spatiotemporal Coding for Local Feature Based Human Action Recognition

**DOI:** 10.1155/2013/405645

**Published:** 2013-09-29

**Authors:** Bin Wang, Yu Liu, Wei Wang, Wei Xu, Maojun Zhang

**Affiliations:** College of Information System and Manage, National University of Defense Technology, 109 Deya Road, Changsha, Hunan 410073, China

## Abstract

We propose a Multiscale Locality-Constrained Spatiotemporal Coding (MLSC) method to improve the traditional bag of features (BoF) algorithm which ignores the spatiotemporal relationship of local features for human action recognition in video. To model this spatiotemporal relationship, MLSC involves the spatiotemporal position of local feature into feature coding processing. It projects local features into a sub space-time-volume (sub-STV) and encodes them with a locality-constrained linear coding. A group of sub-STV features obtained from one video with MLSC and max-pooling are used to classify this video. In classification stage, the Locality-Constrained Group Sparse Representation (LGSR) is adopted to utilize the intrinsic group information of these sub-STV features. The experimental results on KTH, Weizmann, and UCF sports datasets show that our method achieves better performance than the competing local spatiotemporal feature-based human action recognition methods.

## 1. Introduction

Human action recognition in video has been widely studied over the last decade due to its widespread application prospects in the areas such as video surveillance [1, 2], action-based human computer interfaces [3], and video content analysis [4]. It is an important branch in the field of artificial intelligence. It has also been an increasingly active field of computer vision and pattern recognition. However, the action videos are affected by illumination changes, motion blur, occlusion, and other factors. They make human action recognition still a challenging task [5, 6]. 

Many human action recognition techniques have been proposed, and several reviews are devoted to this topic [5, 6]. There are two respects in this field [5]: video representation and classification. Video representation is the process of extracting features from videos and obtaining the behavior representation by encoding the features. Then an action model is learned from the final behavior representations and used to recognize new behaviors. In general, there are two representations methods: global representations [7–14] and local representations [15–25]. Common global representations are derived from silhouettes or body sketch. They need fine foreground segment or body part tracking. Thus, they are sensitive to noise, variations in viewpoint, and partial occlusion. Local representations are based on the local spatiotemporal features together with bag of features (BoF) model. Without foreground segment or body part tracking, they are less sensitive to viewpoint changes, noise, appearance, and partial occlusions [5].

There are three respects in BoF-based human action recognition: extracting local features in videos, obtaining video representation vector via these local features, and classifying action videos with a classifier upon the video representation vector [5]. To obtain video representation vector, several feature coding and pooling methods are provided. Many authors used K-means and vector quantization (VQ) for feature coding, as well as the avg-pooling [16] to group these feature codes to generate the video representation vector. To reduce the quantization error due to K-means and VQ, assign one code word for a feature, soft vector quantization (SVQ) [26] and sparse coding (SC) [27] are adopted to encode local features for action recognition tasks [24]. However, the local features usually reside on nonlinear manifolds [15, 28, 29]. Neither SVQ nor SC can preserve the nonlinear manifold structure. The manifold is nonlinear and not Euclidean in its whole space, but linear and Euclidean in a local region [30, 31]. Because SVQ uses all bases to encode each feature and generates dense codes, it cannot precisely represent the nonlinear manifold structure with a global way. Due to the over complete dictionary, SC tends to choose the code words which are distant to the input features [29]. Thus, it cannot correctly represent manifold data. Hereafter, we consider these limitations both quantization error and loss manifold structure in feature coding as representation error. For this issue, Yu et al. [28] provided a Local Coordinate Coding (LCC) to encode feature with locality-constrained, Wang et al. [29] introduced an improved version of LCC named Locality constrained Linear Coding (LLC) to reduce computational cost, and Wei et al. [32] proposed a local sensitive dictionary learning method for image classification.

**Figure 1:**
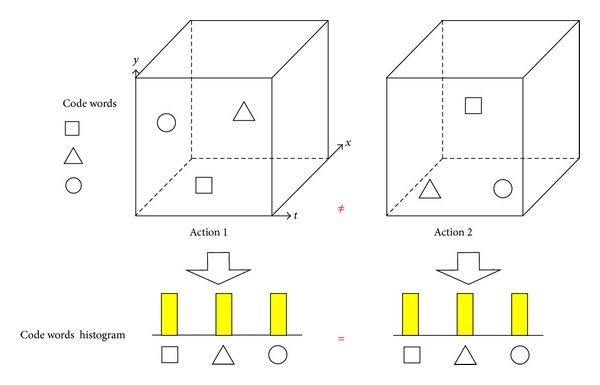
The illustration of that BoF ignores the spatiotemporal relationship of local features in STV. There are two groups of similar features with different spatiotemporal arrangements in STV. They are form Action 1 and Action 2, respectively. However, they cannot be correctly classified with BoF, because it obtains two same code words histograms due to ignoring feature spatiotemporal relationship.

In action classification stage, support vector machine (SVM) has been widely used when the video representation vectors are provided. Recently, inspired by the major success of Sparse Representation-based Classification (SRC) in face recognition [33], some authors [25] explored SRC for human action recognition and achieved better performance than SVM. Nevertheless, these local representation methods suffer one important limitation. They largely ignore the spatiotemporal relationship among local features, such as temporal order and spatial arrangement [34–36]. For example, in Figure 1, two different actions in the left and right space-time-volume (STV) have the same local features and appear different spatiotemporal configurations. Due to the same histograms generated by BoF, they are incorrectly considered as one action. Recently, some researchers exploited some approaches to use spatiotemporal context information [34, 35], local feature distribution [34, 36], and spatial pyramid matching (SPM) [23, 37] with regard to this problem.

**Figure 2:**
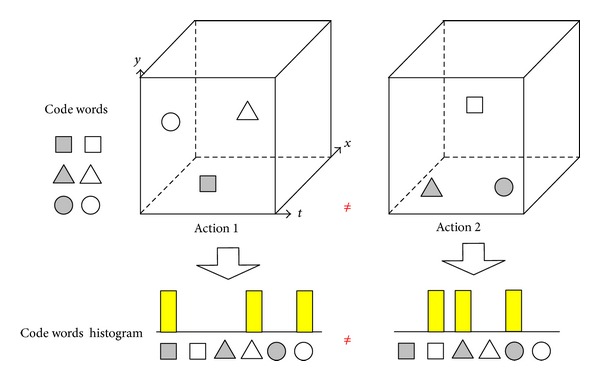
The illustration of our method. The shape of code words indicates appearance information, while the color indicates spatiotemporal position. For example, the code words ■  □ have same appearance but different position. They are considered as two different code words in feature coding. Then, two different code words histograms are obtained for two actions upon these code words. Hence, Actions 1 and 2 which cannot be distinguished in BoF (Figure 1) can be correctly classified with our method.

In this paper, we introduce a Multiscale Locality-Constrained Spatiotemporal Coding (MLSC) method to address this limitation and reduce the representation error simultaneously. To reduce the representation error (quantization error and loss manifold structure), we adopt locality-constraint into dictionary learning and feature coding from the respect of manifold learning [30, 31]. To model the spatiotemporal relationships of local features, we involve feature spatiotemporal positions into dictionary learning and feature coding. Then, the spatiotemporal relationship of local features can be obtained from the features codes. In addition, to handle with the different action styles (the space and time range variant of action), the multiscale spatiotemporal relationship is also modeled by MLSC. In practice, local features are firstly projected into sub space-time-volume (sub-STV) to obtain their spatiotemporal positions. Then dictionary learning and local features coding are implemented with locality and position constraint. To classify one action video (see Figure 2), a group of sub-STV are densely sampled, and a group of sub-STV descriptors are obtained with MLSC and max-pooling [29]. Then Locality-Constraint Group Sparse Representation [38] is adopted for action classification upon these sub-STV descriptors. 

Compared to these methods which use spatiotemporal context information [34, 35] or feature distribution [36] to handle the limitations of BoF, MLSC is a more *fine* and *whole* method, because it records the whole elements (*where, when, who,* and *how*) of local features for human action recognition (detailed in Section 4.6). The experimental results on KTH, Weizmann, and UCF sports datasets show that our method achieves better performance than these methods [23, 34–36] and other local spatiotemporal feature-based methods.

There are three contributions in this paper. First, to solve the limitations of BoF, a novel feature coding method MLSC is proposed for modeling local feature spatiotemporal relationships, at the same time, reducing representation error. In addition, to deal with action style variant, the multiscale spatiotemporal relationship is also modeled by MLSC. Second, to effectively use MLSC, a novel human action recognition framework is proposed (detailed in Figure 2). It extracts the dense sub-STV descriptors from videos and classifies actions upon these descriptors. Third, in order to utilize the intrinsic group information from these sub-STV descriptors within one video, the Locality-Constrained Group Sparse Representation-(LGSR-) [38] based classifier is adopted for action classification.

The rest of this paper is organized as follows. MLSC is proposed in Section 2. The human action recognition framework with MLSC and LGSR is provided in Section 3. Then, experimental results and analysis are shown in Section 4. Finally, conclusions are drawn in Section 5.

## 2. Multiscale Locality-Constrained Spatiotemporal Coding

### 2.1. Modeling Spatiotemporal Relationship with Feature Position

In BoF model, both code words learning and local feature coding only use feature appearing information, but they discard feature position [16]. It is the reason why BoF ignores the feature spatiotemporal relationship. To solve this problem, the feature spatiotemporal positions are involved into dictionary learning and feature coding in this paper. It is inspiring from the work of Liu et al. [39]. They involved spatial locations into DCT-based local feature descriptors to model the spatial relationship of local features for face recognition. Their experimental results showed that feature locations can improve local feature-based face recognition accuracy. 

In this paper, the feature descriptor and feature spatiotemporal location (*x*, *y*, *t*) are connected together to generate a new feature descriptor **f** ^*α*,*β*^:

(1) $$\begin{array}{c} f^{\alpha ,\beta }={\left[\text{HO}{\text{G}}^{T},\text{HO}{\text{F}}^{T},\alpha \left(x,y\right),\beta t\right]}^{T}, \end{array}$$

where *α* and *β* are the position weighting factors which represent the importance of the spatial and temporal position in feature matching, respectively. The histograms of gradient orientations (HOG) and the histogram of optic flow (HOF) [20] are adopted. Then the feature spatiotemporal relationship can be modeled with dictionary learning and feature coding upon the new feature descriptor **f** ^*α*,*β*^. 

To easily explain the role of involving feature position into dictionary learning and feature coding, we adopt K-means to learn dictionary and VQ to encode features, respectively. The representation error caused by them will be solved in Section 2.2. **D** ^*α*,*β*^ ∈ *R* ^*N*×*M*^ is a dictionary learnt with K-means clustering upon the features **F** ^*α*,*β*^ = [**f**_1_^*α*,*β*^,…, **f**_*n*_^*α*,*β*^]. In **D** ^*α*,*β*^, each visual words **b** ^*α*,*β*^ has three types information: visual words appearing information (HOG/HOF), spatial position (*x*, *y*), temporal position (*t*). The code **c** for feature **f** ^*α*,*β*^ is obtained with VQ:

(2) $$\begin{array}{c} c_{i}=\{\begin{array}{cc} 1, & \text{if}\;i=\underset{i}{\arg\;\min}{||b_{i}^{\alpha ,\beta }-f^{\alpha ,\beta }||}_{2},\\ 0, & \text{otherwise}, \end{array} \end{array}$$

where **f** ^*α*,*β*^ is the input feature and is described with (1). **b**_*i*_^*α*,*β*^ is the *i*th base in dictionary **D** ^*α*,*β*^. **c** ∈ *R* ^*M*^ is the code for **f** ^*α*,*β*^.

According to (1), the base **b** ^*α*,*β*^ which is chosen to encode **f** ^*α*,*β*^ must be the closest to **f** ^*α*,*β*^ in three respects: feature similarity, spatial distance, and temporal distance. Thence, the spatiotemporal position of **f** ^*α*,*β*^ form its code **c** can be obtained. Given a group of local features, their spatiotemporal relationship can be represented with their code words histogram:

(3) $$\begin{array}{c} H_{i}=\frac{1}{n}\sum_{i=1}^{n}C_{i}, \end{array}$$

where **H** ∈ *R* ^*M*^ is the code words histogram, *n* is the number of features, and **C** is the code of these features.

For example, as illustrated in Figure 2, these two actions in Figure 1 can be distinguished with their new histograms. Benefiting from involving feature position into code words, two different code words histograms are provided for Actions 1 and 2. Actions that have similar features but different spatiotemporal relationship can be correctly classified by this method. Therefore, involving spatiotemporal position into dictionary learning and feature coding is a feasible way to model the spatiotemporal relationship of features for human action recognition.

### 2.2. Reducing Representation Error with Locality Constraint

In Section 2.1, K-means and VQ are adopted in dictionary learning and feature coding. However, Yu et al. [28] discovered that VQ cannot handle nonlinear manifold structure well. Because it is a 0th order (constant) approximation of object functions from the view of function approximation. In addition, VQ causes nontrivial quantization error. They suggested that 1st-order (linear) approximation can solve these problems and introduced adding locality constraint into object function:

(4) $$\begin{array}{c} c=\underset{c}{\arg\;\min}{||f^{\alpha ,\beta }-D^{\alpha ,\beta }c||}_{2}^{}+\lambda {||p⊙c||}_{1},\;\text{st:}{\;1}^{T}c=1, \end{array}$$

where the first term represents the reconstruction error of an input feature **f** ^*α*,*β*^ with respect to dictionary **D** ^*α*,*β*^, the second term is locality-constraint regularization on code **c**, and *λ* is a regularization factor to balance these terms. In the second term, **p**_*j*_ = ||**f** ^*α*,*β*^−**b**_*j*_^*α*,*β*^||_2_ is the distance between **f** ^*α*,*β*^ and *j*th code word **b**_*j*_^*α*,*β*^, ⊙ is the element product, and 1^*T*^ **c** = 1 is the shift invariant constraint according to [28]. 

Equation (4) tends to choose the code words which are close to **f** ^*α*,*β*^for generating the code **c**. Because **p** is fixed, to minimize ||**f** ^*α*,*β*^−**D** ^*α*,*β*^ **c**||_2_ + *λ*||**p**⊙**c**||_1_, one needs to make the coefficient **c**_*j*_ corresponding to large **p**_*j*_ equals 0. In addition, ||||_1_ is spares regularization term and intends to obtain sparse solution. Sparsity indicates that many elements in **c** are zero, while only a few are nonzero. Thus only a few code words near to **f** ^*α*,*β*^ are selected to encode feature **f** ^*α*,*β*^. Obviously, the selected code words belong to the local neighbor of **f** ^*α*,*β*^.

However, an iterative optimization is needed to solve the *l* ^1^optimization problem in (4). To reduce the computational cost in (4), we use ||**p**⊙**c**||_2_ to replace ||**p**⊙**c**||_1_. Consider

(5) $$\begin{array}{c} c=\underset{c}{\arg\;\min}{||f^{\alpha ,\beta }-D^{\alpha ,\beta }c||}_{2}^{}+\lambda {||p⊙c||}_{2}^{},\;\text{st:}\;1^{T}c=1. \end{array}$$

In (5), **p** is fixed. To minimize ||**p**⊙**c**||_2_, the code words far from **f** ^*α*,*β*^ will be assigned zero in **c**. In contrast, the code words near to **f** ^*α*,*β*^ will be assigned nonzero in **c**. Therefore, similar to (4), the code words that belong to the neighbor of **f** ^*α*,*β*^ will be selected to encode **f** ^*α*,*β*^. From the respect of manifold learning [23, 25], although the whole data of a manifold are nonlinear and Euclidian, in a local region, they can be considered as linear [23–25]. Therefore, benefiting from the locality constraint, the problems of VQ can be solved.

The object function in (5) can be solved with an analytical solution according to [32]:

(6) $$\begin{array}{c} \rho ={\left(\psi +\lambda \;\mathrm{diag}{\left(p_{j}\right)}^{2}\right)}^{-1}1,\\ \psi ={\left(f^{\alpha ,\beta }1^{T}-D\right)}^{T}\left(f^{\alpha ,\beta }1^{T}-D\right),\\ c=\frac{\rho }{1^{T}\rho }. \end{array}$$

Similarly, the problems of K-means dictionary learning can also be solved with locality constraint. According to [35], the object function of our dictionary learning method is formulated as follows:

(7) min⁡Dα,β,C||Fα,β−Dα,βC||2+λ∑i=1n||pi⊙ci||2, st: 1Tci=1∀i=1,…,n,

where **F** ^*α*,*β*^ = {**f**_1_^*α*,*β*^,…, **f**_*n*_^*α*,*β*^}, *n* is the number of input local features, **c**_*i*_ ∈ *R* ^*M*^ is the *i*th column of **C**, and **p**_*i*_ ∈ *R* ^*M*^ is the locality adaptor whose *j*th element is given by **p**_*ij*_ = ||**f**_*i*_^*α*,*β*^−**d**_*j*_^*α*,*β*^||_2_. Equation (7) can be effectively solved with the Locality-Sensitive Dictionary Learning (LSDL) in [32]. 

### 2.3. Modeling the Multiscale Spatiotemporal Relationship of Local Features

Due to the different styles of human action, it is difficult to model the spatiotemporal relationship of local features in a single space-time scale. The actions with different styles appear in different motion range (spatial scale is different) and speed (temporal scale is different). Therefore, it is necessary to capture their multiscale spatiotemporal relationship in feature coding. In implementation, instead of building spatial or temporal pyramid structures, we use position weighting factors *α* and *β* to control the spatial and time scales, respectively. According to (1), a large (small) *α* or *β* intends to select the code words from a small (large) spatial or temporal neighbor. Thus we can adjust *α* or *β* to obtain the multiscale feature descriptor **f** ^ms^:

(8) $$\begin{array}{c} f^{\text{ms}}=\left\{f^{\alpha (1),\beta (1)},\ldots ,f^{\alpha (i),\beta (j)},\ldots \right\}, \end{array}$$

where *i* ∈ [1, *n*_*s*_], *j* ∈ [1, *n*_*t*_], and *n*_*s*_ and *n*_*t*_ are the numbers of spatial and time scales, respectively. For example, we set *α* = {4,3, 2,1}, *β* = {4,3, 2,1}.

Then the code is given as

(9) $$\begin{array}{c} c^{\text{ms}}={\left[{\left(c^{\alpha (1),\beta (1)}\right)}^{T},\ldots ,{\left(c^{\alpha (i),\beta (j)}\right)}^{T},\ldots \right],}^{T}\\ c^{\alpha (i),\beta (j)}=\underset{c}{\arg\;\min}{||f^{\alpha (i),\beta (j)}-D^{\alpha (i),\beta (j)}c||}_{2}^{}+\lambda {||p⊙c||}_{2}^{}, \end{array}$$

where **D** ^*α*(*i*),*β*(*j*)^ is the dictionary learnt by LSDL with the features descriptors at *i*th spatial and *j*th temporal scales. 

## 3. Human Action Recognition with MLSC and LGSR

### 3.1. Framework

The spatiotemporal positions (*x*, *y*, *t*) of local features play a key role in our method. Intuitively, we can construct spatial coordinate system with human ROI and build time coordinate system with a complete action cycle. Profiting from the existing methods of extracting human ROI from videos, the space coordinate system is easy to be set up. Because it is difficult to estimate action cycles in videos, the time coordinate system is difficult to establish. Fortunately, the feature spatiotemporal relationships can be locally modeled by sub-STV. We propose a novel framework without estimating action cycles as follows: (1) it densely samples several sub-STV from one video; (2) it carries out MLSC in each sub-STV to obtain a sub-STV descriptor; and (3) it classifies action upon these sub-STV descriptors with LGSR. 

**Figure 3:**
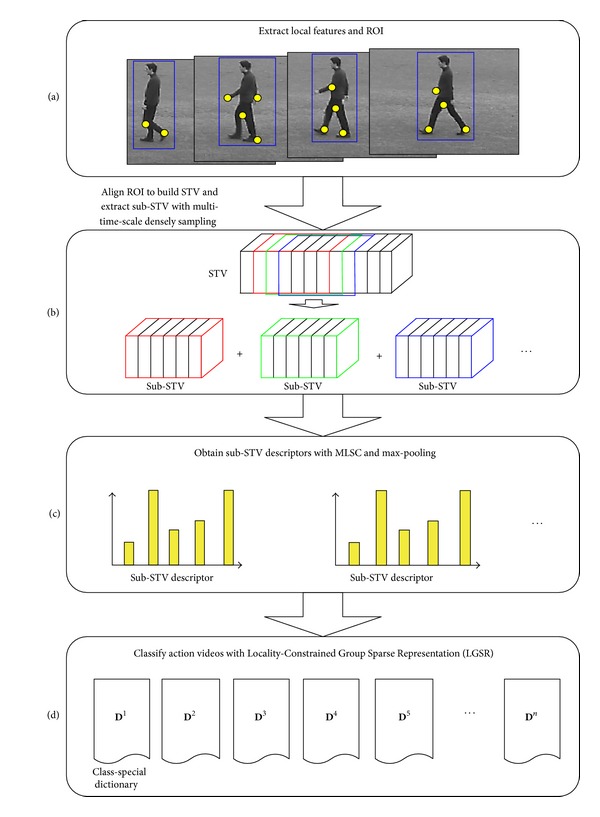
The flow chart of human action recognition with MLSC and LGSR.

The proposed framework is illustrated in Figure 3. In the first step (Figure 3(a)), the local features such as space time interest points (STIP) and human ROI are extracted in each action video. In the second step (Figure 3(b)), it aligns ROI to build STV and extract sub-STV with multitime-scale densely sampling (detailed in Section 3.2). Then, many sub-STV in one video will be collected. In the third step (Figure 3(c)), it obtains a group of sub-STV descriptors with MLSC and max-pooling (detailed in Section 3.3). In the last step (Figure 2(d)), it utilizes these sub-STV descriptors to classify action with LGSR (detailed in Section 3.4).

### 3.2. Extract Sub-STV with Multi-Time-Scale Densely Sampling

The feature spatiotemporal relationships in STV are locally captured with the multi-time-scale densely sampling (MTDS) method. First, a set of time scales is defined for MTDS according to the possible action cycle lengths. Several sub-STVs are then densely sampled by a sliding window operation with one frame step (Figure 3(b)). Finally, several space time coordinate systems are established based on these sub-STVs. After that, a group of multiscale feature descriptors are obtained with (8) with setting *β* = {*a*, *a*, *a*, *a*} (*a* is a constant), because the multi-time-scale information has been considered in MTDS. 

The advantage of MTDS is that it is not necessary to consider whether the time coordinate system is aligned with the human action cycles in each sub-STV. Because if only the training samples are sufficient, then any tests sub-STV can always find a matching sub-STV in the training samples. Usually, this condition can be satisfied in real applications. 

### 3.3. Describe Sub-STV with MLSC and Max-Pooling

Given a sub-STV, we take it as the space-time coordinate system (*x*, *y*, *t*) to generate a group of multiscale feature descriptors:

(10) $$\begin{array}{c} F^{\text{ms}}=\left\{f_{1}^{\text{ms}},\ldots ,f_{n}^{\text{ms}}\right\}. \end{array}$$

Then, we use MLSC to encode each feature and obtain multiscale codes:

(11) $$\begin{array}{c} C^{\text{ms}}=\left[c_{1}^{\text{ms}},\ldots ,c_{n}^{\text{ms}}\right]. \end{array}$$

After coding each feature, we use the max-pooling [29] to get the sub-STV descriptor:

(12) $$\begin{array}{c} s\left(j\right)=\max\left\{c_{1}^{\text{ms}}\left(j\right),\ldots ,c_{n}^{\text{ms}}\left(j\right)\right\}, \end{array}$$

where **s**(*j*) is *j*th element in this sub-STV descriptor **s** and **c**_*i*_^ms^ is the MLSC coefficient vector for *i*th feature.

### 3.4. LGSR-Based Action Videos Classification

To utilize the intrinsic group information from these sub-STV descriptors within one video for action classification, we adopt the locality-Constrained Group Sparse Representation (LGSR) to classify actions. LGSR was proposed in [39] for human gait recognition. It is an extended sparse representation-based classifier (SRC). The pioneering work of SRC was proposed in [33] and used to classify face images by minimizing the norm-regularized reconstruction error. There are three advantages of LGSR comparing with SRC: (1) SRC is designed for single image classification and cannot directly classify a group of samples, while LGSR is designed for sample group classification; (2) the locality constraint in LGSR is more reasonable than sparsity constraint in SRC, especially for representing manifold data [29, 32]; (3) LGSR is a block sparse constraint classifier. It is better than SRC in classification task when the used features are discriminative. The comparison experiment in Section 4.5 also proves that LGSR is more suitable than SRC for our task.

The object function of LGSR is defined as

(13) $$\begin{array}{c} A^{*}=\underset{A}{\arg\;\min}\left(\frac{1}{2}{||S-DA||}_{F}^{2}+\lambda \sum_{k=1}^{K}{||P^{k}⊙A^{k}||}_{F}\right), \end{array}$$

where the first term represents the reconstruction error of the test action with respect to all the actions. The second term is the weighted mixed-norm-based regularization on the reconstruction coefficient **A**. *λ* is the regularization parameter to balance these terms. **D** is the classification dictionary constructed by connecting *K* class-special dictionaries [**D** ^1^,…, **D** ^*K*^]. Each class-special dictionary **D** ^*k*^ is learnt with LSDL [32] from the sub-STV descriptors corresponding to the *k*th action. **S** is the group of sub-STV descriptors for one test action. **A** ^*k*^ is one part of **A** and corresponds to **D** ^*k*^. **P** ^*k*^ is the distance matrix between **S** and **D** ^*k*^. The *i*th and *j*th element in **P** ^*k*^ is calculated as **P**_*ij*_^*k*^ = ||**S**_*i*_−**D**_*j*_^*k*^||_2_. Since **A** ^*k*^ values are independent of each other, we can separately update each **A** ^*k*^ using its subgradient [36]. To solve (14), the active set-based subgradient descent algorithm in [38, 39] was employed.

 Once the optimal reconstruction coefficient **A** is obtained, maximum weighted inverse reconstruction error (maxWIRE) criterion [38] is adopted for action classification. It is better than the original minimum reconstruction Error (minRE) criterion in [33].

WIRE is defined as

(14) $$\begin{array}{c} \text{WIRE}\left(k\right)=\frac{{||A^{*k}||}_{F}^{}}{{||S-D^{k}A^{*k}||}_{F}^{}}. \end{array}$$

The action video label *L* is decided with the maximum WIRE:

(15) $$\begin{array}{c} L^{*}=\underset{k}{\arg\;\max}\left(\text{WIRE}\left(k\right)\right). \end{array}$$

## 4. Experiment and Analysis 

In this section, the effectiveness of our MLSC is evaluated on three public datasets: Weizmann, KTH, and UCF sports. The leave-one-out cross-validation (LOOCV) is used to evaluate the performance of our algorithm. It employs actions from some people as the test samples, meanwhile leaving the remaining actions from other people as the training samples.

### 4.1. Experiment Setup

In all experiments, cuboids [16] is adopted to extract spatiotemporal local features, and HOG/HOF [20] is adopted to describe these features. According to [16], the standard space scale value 3 and time scale value 2 are used in cuboids detector. To extract ROI, we label a bounding box for the actor that locates at the first frame in each split and then track actor to obtain the ROI for KTH dataset; the annotation bounding boxes are used for extracting ROI for UCF sports dataset, and a rotation operation is used to obtain oriented ROI; and the background subtraction results are used for the Weizmann dataset. To capture multiscale temporal relationship of local features, the lengths of sub-STV are set as 5, 10, 25, and 50 frames. To capture multiscale spatial relationship of local features, four spatial scales are used. The parameters are set as *α* = {4,3, 2,1} and *β* = {1,1, 1,1}. In MLSC, the dictionary size is set to 1000. Since there are 4 spatial scales, the dimension of a sub-STV descriptor is 4000. In order to guarantee that the class-special dictionaries in LGSR are over complete, PCA is adopted to reduce the dimension of the sub-STV descriptor to 400. In LGSR, the size of each class-special dictionary is set to 800. The other parameters in our methods (for example *σ* and *λ*) and the parameters of other methods are evaluated by 5-fold cross-validation.

### 4.2. Datasets

**Figure 4:**
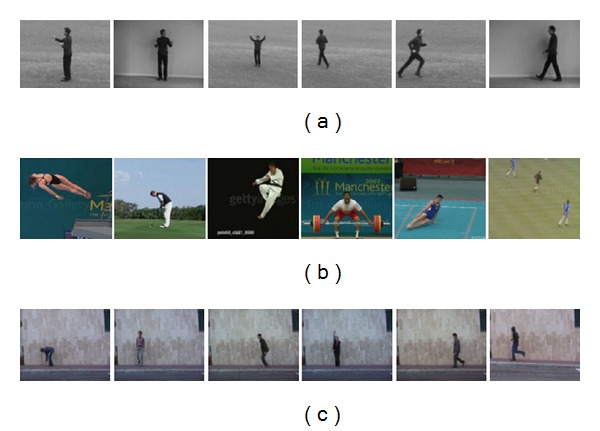
Examples from the public datasets: (a) KTH dataset; (b) UCF sports dataset; (c) the Weizmann dataset.

The KTH dataset contains six types of human action examples (i.e., boxing, hand clapping, hand waving, jogging, running, and walking) featuring 25 different subjects. Each action is performed in four scenarios: indoors, outdoors, outdoors with scale variation, and outdoors with different clothes. Overall it has 599 low-resolution video clips (160 × 120 pixels), for one of the videos is missing. Examples of this datasets can be seen in Figure 4(a). 

UCF sports dataset includes a set of 150 videos, which are collected from various broadcast sports channel such as BBC and ESPN. It contains 10 different actions: diving, golf swing, horse riding, kicking, lifting, running, skating, swing bar, swing floor, and walking. This dataset is challenging with a wide range of scenarios and viewpoints. Examples of this datasets can be seen in Figure 4(b).

Weizmann: this dataset contains 93 low-resolution video clips (180 × 144 pixels) from nine different subjects, each of whom performs 10 different actions including walking (walk), running (run), jumping (jump), galloping sideways (side), bending (bend), one-hand-waving (wave one), two-hands-waving (wave two), jumping in place (pjump), jumping jack (jack), and skipping (skip). One of the subjects performs walking, running, and skipping twice. The camera setting is fixed and there is no occlusion or viewpoint change. Besides, each subject performs under similar plain background. Some examples are demonstrated in Figure 4(c). 

### 4.3. Comparing with BoF

BoF-based action representation methods together with existing local feature coding methods VQ, SC, LLC [29], and our MLSC are further compared under the same condition that K-nearest Neighbor (KNN) classifier is used in classification stage. In KNN, *k* is set to 5. Keeping the same dictionary size with MTSC, K-means clustering is used to learn dictionary for VQ and LLC, and the software in [40] is adopted for SC. In LLC, the locality constraint parameter *k* is set to 5. In our methods, a group of sub-STVs descriptors are extracted from one test video and classified with KNN. After that the vote scores of these sub-STVs are used to label this test video. In feature pooling phase, avg-pooling is used for VQ, while max-pooling is adopted for SC, LLC, and MLSC. 

In addition, to evaluate these factors which are used to improve BoF from feature coding in Section 2, another comparison is carried out. First, considering the feature position constraint in Section 2.1, the coding method in (2) is considered as the basic spatiotemporal coding (StC). Second, considering the locality constraint in Section 2.2, the coding method in (5) is considered as the locality-constrained spatiotemporal coding (LSC). In this comparison experiment, the dictionary size is still set to 1000. K-means clustering is adopted to learn dictionary for StC. LSDL is adopted to learn dictionary for LSC. Avg-pooling is used for StC, and max-pooling is adopted for LSC. The parameter for KNN is set to 5. The spatial and temporal control factors are set as *α* = {4,3, 2,1} and *β* = {1,1, 1,1}. 

The results of comparison are shown in Table 1. There are the average recognition accuracies on three datasets. The basic spatiotemporal coding method (StC) achieves better performance than VQ, SC, and LLC. This demonstrates that considering the spatiotemporal relationship is important for human action recognition in video. The locality-constrained spatiotemporal coding is better than StC. In addition, the locality constraint is useful to handle the manifold of local features. Finally, benefiting from modeling the multiscale spatiotemporal relationship of local features, MLSC achieves the highest average recognition accuracy on each dataset.

### 4.4. MLSC versus SPM

**Figure 5:**
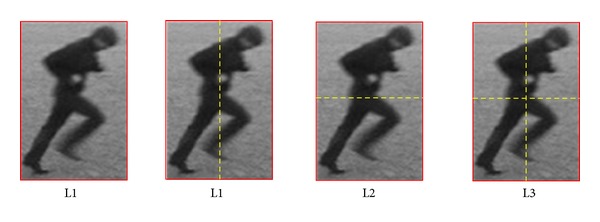
Confusion matrix for KTH dataset of our methods LGSR and MLSC.

The spatial pyramid match (SPM) model has been adopted to capture the spatial relationships of local spatiotemporal features [23]. Here, a 4-level SPM (detailed in Figure 5) is used for evaluation. MLSC and SPM, LLC, and Max-pooling are employed to describe sub-STV, respectively. KNN classifier is also used to classify sub-STVs. The vote score-based classifier (similar with Section 4.3) is adopted to label a test video.  Table 2 shows the average recognition accuracies. MLSC achieves better performance than SPM on all datasets. Different from SPM [37] which only considers the spatial relationship of local features, MLSC simultaneously considers the spatial and temporal relationships. In addition, comparing with the fixed grids used in SPM, MLSC is a more flexible representation.

### 4.5. LGSR versus SRC

To prove the ability of using LGSR for action classification, the standard SRC [29] is also evaluated. The object function of SRC is defined as

(16) $$\begin{array}{c} A_{i}^{*}=\underset{A_{i}}{\arg\;\min}{||S_{i}-DA_{i}||}_{2}^{}+\omega {||A_{i}||}_{1}, \end{array}$$

where **S**_*i*_ is the *i*th sub-STV descriptor in **S**, **A**_*i*_ is its corresponding code. Similar to LGSR, the maxWIRE criterion is also used in SRC. As mentioned in Section 3.4, there are three advantages of LGSR comparing with SRC. In particular, if the features are not shared with other classes, the block sparse constraint is more suitable for the classification than sparse constraint. Hence LGSR is relatively better than SRC for classification task when using less shared features. The comparison results with average accuracy (Table 3) show that LGSR achieves better performance than SRC on KTH and UCF sports datasets. It is worth to note that Guha and Ward [25] suggested that sparse constraint is more important than block sparse constraint in human action recognition based on local spatiotemporal features. Comparing with local spatiotemporal features, the obtained sub-STV descriptors with MLSC are less shared with other actions. Hence, it is better to utilize block sparse constraint than sparse constraint for action classification together with MLSC.

### 4.6. Comparing with Other Methods

**Figure 6:**
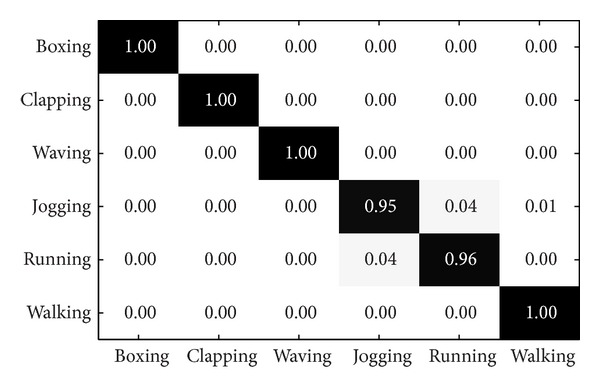
Confusion matrix for UCF sports dataset of our methods LGSR and MLSC.

**Figure 7:**
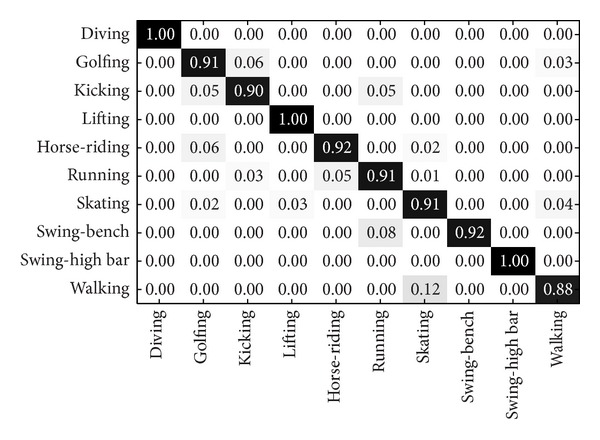
4-level Spatial Pyramid Match (SPM).

The present and some previously published results are compared in Table 4. The experiment setting “split” means that it randomly selects some people for training and leaves others for testing. The competing methods include local representation-based methods [15, 21, 24, 25, 34–36], global representation-based methods [12]. In detail, SC was used for feature coding together with BoF in [24], and a new local feature detector was proposed for human action recognition in [21], local feature distribution information was used in [36], spatiotemporal context feature was employed in [34], a spatiotemporal context constraint coding method was utilized in [35], sparse representation-based classification methods was applied in [25], and the global representation method was adopted in [12]. It demonstrates that our method achieves better performance than the competing methods. The confusion matrices for KTH and UCF sports datasets of our method LGSR+MLSC are shown in Figures 6 and 7, respectively.

First, benefiting from involving spatiotemporal locations into code words learning and feature coding, our method performs better than these methods [15, 24, 25] which only use the feature appearance information to represent human action. Second, comparing with the feature distribution feature [36] and spatiotemporal context methods [34, 35], our method is a *fine* and *whole* method. For example, as illustrated in Figure 2, each local feature has four types of information (*where, when, who,* and *how*) in STV. First, the coordinate (*x*, *y*) and (*t*) indicates *where* and *when* the body part appears respectively. Second, the feature appearance (described as HOG) indicates *who* (which human body part). Third, the motion information (described as HOF) indicates *how* this body part moves. In MLSC, all of these pieces information (*where, when, who, *and* how*) have been modeled with involving (*x*, *y*), (*t*), HOG, and HOF into feature coding. However, these methods [34–36] ignore some one of these information (*where, when, who, *and* how*) in action representation processing. Hence, it implies that our method records the whole elements (*where,  when,  who,* and *how*) of local features for human action recognition.

## 5. Conclusion

In this paper, in order to capture the spatiotemporal relationships of local spatiotemporal features for human action recognition task, we encode feature appearance and spatiotemporal position information together with locality constraint. The experimental results show that (1) feature spatiotemporal position is effective for action recognition (2) involving feature position into feature coding is a beneficial alternative way for this task. In particular, it is a better approach than feature distribution [36], spatiotemporal context [35], and SPM-based methods [23], when using a multiscale version. 

The major limitation is that human ROI is required to construct STV. Although, dissimilar to the global representation methods which need the fine foreground segmentation, the coarse human box is enough in our method. It is valuable to explore new methods to capture the spatiotemporal location of local features in our future work.

## Figures and Tables

**Table 1:** The average accuracy of BoF together with VQ, SC, LLC and our methods on KTH, Weizmann, UCF sports datasets (unit: %).

Methods	KTH	UCF sports	Weizmann
KNN + BoF + VQ	86.5	75.5	88.1
KNN + BoF + SC	88.1	78.1	89.6
KNN + BoF + LLC	89.5	79.7	90.8
KNN + StC (α = 4)	89.1	78.6	**91.7**
KNN + StC (α = 3)	**90.2**	79.6	90.1
KNN + StC (α = 2)	88.4	**81.4**	89.5
KNN + StC (α = 1)	87.9	79.8	88.7
KNN + LSC (α = 4)	90.5	80.5	92.8
KNN + LSC (α = 3)	**91.8**	82.1	**92.1**
KNN + LSC (α = 2)	91.1	**83.5**	91.8
KNN + LSC (α = 1)	90.9	82.5	91.2
KNN + MLSC	**94.4**	**85.6**	**94.9**

**Table 2:** Comparison results between MLSC with SPM (UNIT: %).

Methods	KTH	UCF sports	Weizmann
KNN + SPM	91.7	82.8	93.5
KNN + MLSC	94.4	85.6	96.5

**Table 3:** Comparison results between LGSR and SRC (UNIT: %).

Methods	KTH	UCF sports	Weizmann
SRC + MLSC	96.5	92.1	100
LGSR + MLSC	**98.5**	**93.5**	**100**

**Table 4:** Comparison results with other methods (unit: %).

Methods	Year	Experiment setting	KTH	UCF sports	Weizmann
Zhu et al. [24]	2010	Split	94.92	84.33	—
Wu et al. [34]	2011	LOOCV	94.5	91.3	—
Escobar and Kornprobst [21]	2012	Split	90.56		99.26
Guha and Ward [25]	2012	LOOCV	—	91.1	98.9
Bregonzio et al. [36]	2012	LOOCV	94.33	—	96.66
Zhang et al. [35]	2012	LOOCV	95.06	87.33	—
Saghafi and Rajan [12]	2012	LOOCV	92.6	—	100
Deng et al. [15]	2013	LOOCV	96.91	88.4	100
LGSR + MLSC		LOOCV	98.5	93.5	100
